# Si-based agent alleviated small bowel ischemia–reperfusion injury through antioxidant effects

**DOI:** 10.1038/s41598-024-54542-7

**Published:** 2024-02-20

**Authors:** Masato Shimada, Yoshihisa Koyama, Yuki Kobayashi, Yasunari Matsumoto, Hikaru Kobayashi, Shoichi Shimada

**Affiliations:** 1 Department of Neuroscience and Cell Biology, Osaka University Graduate School of Medicine, 2-2 Yamadaoka, Suita, Osaka, 565-0871 Japan; 2 Addiction Research Unit, Osaka Psychiatric Research Center, Osaka Psychiatric Medical Center, Osaka, 541-8567 Japan; 3 Global Center for Medical Engineering and Informatics, Osaka University, Suita, 565-0871 Japan; 4 Integrated Frontier Research for Medical Science Division, Institute for Open and Transdisciplinary Research Initiatives (OTRI), Osaka University, Suita, 565-0871 Japan; 5 SANKEN, Osaka University, Osaka, 567-0047 Japan

**Keywords:** Gastrointestinal diseases, Experimental models of disease

## Abstract

The progression of small bowel ischemia–reperfusion (IR) injury causes cells in the intestinal tract to undergo necrosis, necessitating surgical resection, which may result in loss of intestinal function. Therefore, developing therapeutic agents that can prevent IR injury at early stages and suppress its progression is imperative. As IR injury may be closely related to oxidative stress, antioxidants can be effective therapeutic agents. Our silicon (Si)-based agent, an antioxidant, generated a large amount of hydrogen in the intestinal tract for a prolonged period after oral administration. As it has been effective for ulcerative colitis, renal failure, and IR injury during skin flap transplantation, it could be effective for small intestinal IR injury. Herein, we investigated the efficacy of an Si-based agent in a mouse model of small intestinal IR injury. The Si-based agent suppressed the apoptosis of small intestinal epithelial cells by reducing the oxidative stress induced by IR injury. In addition, the thickness of the mucosal layer in the small intestine of the Si-based agent-administered group was significantly higher than that in the untreated group, revealing that Si-based agent is effective against small intestinal IR injuries. In the future, Si-based agents may improve the success rate of small intestine transplantation.

## Introduction

The small intestine has a variety of functions, including the absorption of vitamins and trace elements involved in their metabolism, in addition to the absorption of essential nutrients, such as amino acids, glucose, and fatty acids, and plays a role in maintaining homeostasis in the body. Moreover, the small intestine is consistently subjected to various external factors, including digestive enzymes and intestinal bacteria, making it susceptible to continuous exposure. Therefore, the small intestine uses two mechanisms to maintain a stable intestinal environment. One is a physical defense by mucus composed of mucin and cell adhesion of small intestinal epithelial cells, and the other is a chemical defense by antibacterial peptides such as lactoferrin and lysozyme produced by small intestinal epithelial cells^1^. In contrast, recent research suggested that systemic inflammatory diseases such as rheumatoid arthritis^2^ and systemic lupus erythematosus^3^ develop or exacerbate when changes in the composition of the intestinal flora occur due to disturbances in the intestinal environment. Therefore, the importance of maintaining intestinal function has been confirmed.

However, the small intestine is considered to be one of the most vulnerable organs among many organs. Even today, when organ transplantation technology is developing, small bowel transplantation remains difficult, and the number of cases of small bowel transplantation is significantly lower than that of other organs^4^. Factors related to the fragility of the small intestine include rejection after organ transplantation and low resistance to organ necrosis due to ischemia–reperfusion (IR) injury^4^. Tissue vulnerability to IR injury of the small intestine is a problem not only in organ transplantation but also in diseases with a relatively high frequency in clinical settings such as acute mesenteric arterial occlusion and strangulating intestinal obstruction^5^. When a revascularization procedure is performed after treatment for these diseases, IR injury is an inevitable complication. In the worst case, intestinal necrosis occurs, resulting in a poor prognosis. Once intestinal necrosis has occurred, surgical resection of the intestine is the only therapeutic option available. Therefore, preventing the progression of IR injury at an early stage is an urgent issue.

A major cause of IR injury is oxidative stress caused by reactive oxygen species (ROS) generated by various mechanisms, including mitochondrial respiratory chain injury^6,7^. Antioxidants reduce IR injury by scavenging ROS and are effective therapeutic agents for suppressing intestinal necrosis.

Previous studies have shown that the administration of antioxidants such as hydrogen^8^ and polyphenols^9–11^ alleviated IR injury. Hydrogen is an excellent antioxidant that specifically scavenges harmful ROS, such as highly toxic hydroxyl radicals^12^.

Our silicon (Si)-based agent reacts with water to generate a large amount of hydrogen^13^. More hydrogen was produced when it reacted with a weakly alkaline solution than when it reacted with a neutral solution. In addition, it is expected to be an effective novel antioxidant against diseases associated with oxidative stress because it stably produces a large amount of hydrogen in the intestine after oral administration^14^. In fact, Si-based agent has been shown to alleviate the symptoms of diseases such as ulcerative colitis^15^, intestinal disease, IR injury during skin flap transplantation^16^, and renal IR injury^17^. Therefore, Si-based agents may suppress IR injury to the small intestine. In this study, we investigated the efficacy of an Si-based agent against small intestinal IR injury using a mouse model of IR injury for superior mesenteric artery occlusion.

## Results

### Si-based agent alleviated the damage to the small intestinal epithelium caused by ischemia–reperfusion injury

**Figure 1:**
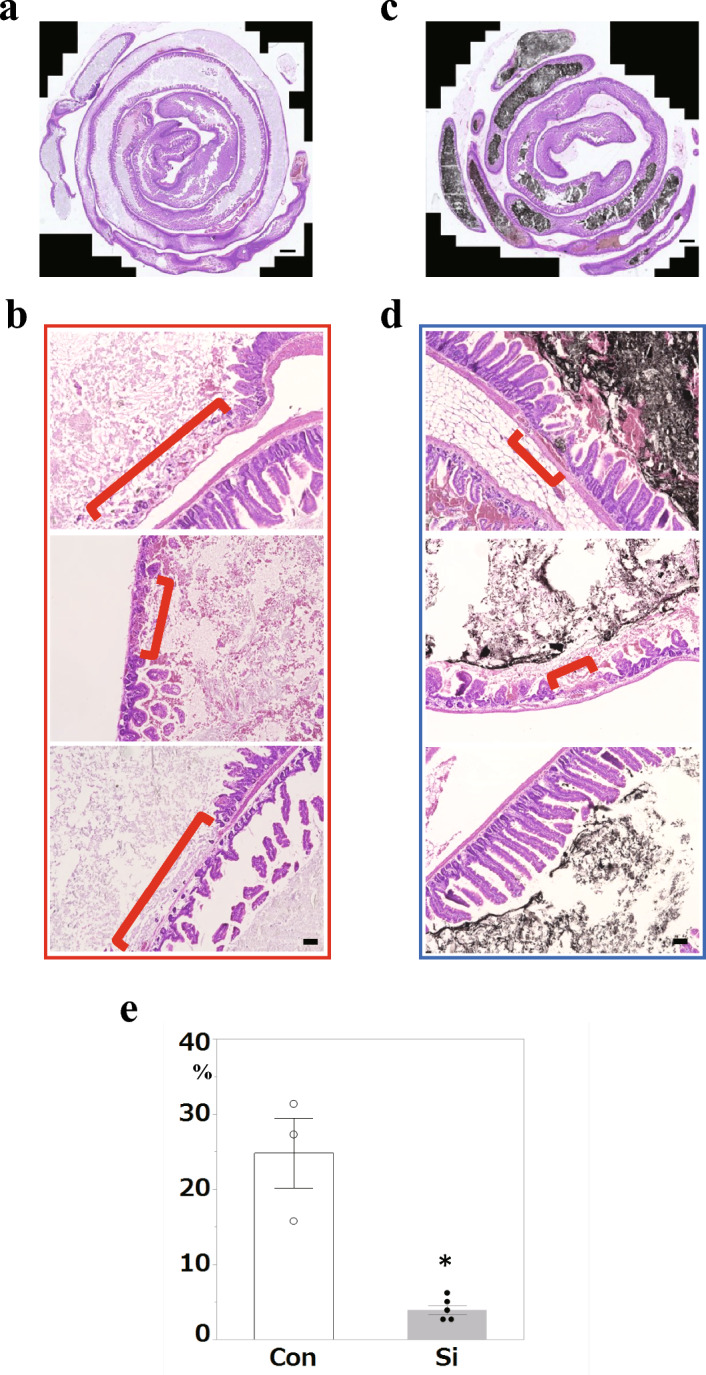
Pathological analysis of mouse small intestine using HE-stained specimens. (**a**–**d**) The representative microphotographs of the hematoxylin and eosin (HE)-stained small intestine in the control (Con) group (**a**, **b**) and silicon-treated (Si) group (**c**, **d**). (**a**, **c**) Whole small intestine and (**b**, **d**) lesion site of the small intestine. Red parentheses: lesion site. Scale bar: 200 µm (**a**, **c**), 40 µm (**b**, **d**). (**e**) The measured results of the lesion area in the small intestine. Bar chart indicates the mean values. White: Con group. Grey: Si group. Data are expressed as mean. Standard error of the mean (SEM) of three (Con) or five mice (Si) per group. *p < 0.05 versus Con group, determined by Student’s paired *t*-test.

To investigate whether Si-based agents reduced the injury of the small intestine tissue caused by IR, we performed a pathological analysis of the small intestine after IR injury and then investigated the entire damaged area of epithelial cells caused by apoptosis, necrosis, and physical injury using the Swiss-Roll method small intestine specimens. In both the Si-treated group (Si group) and the untreated control group (Con group), many epithelial cells at the tip of the villi protruding into the lumen were shed; however, the slough was limited to the villi. Moreover, ulcers damaging the submucosal layer were not observed. First, the width of the damaged area of the villi was compared between the two groups. In the Con group, villi were absent in a wide range from the jejunum to the ileum, and disruption of the villus structure was observed in many regions (Fig. 1a, b). In contrast, the Si group showed a much more localized villus defect (Fig. 1c, d). The black material in the intestinal lumen was the Si-based agent. Statistical analysis of the difference in the spread of the damaged area between the two groups showed that the tissue damaged area in the Si group was significantly smaller than that in the Con group (Fig. 1e).

**Figure 2:**
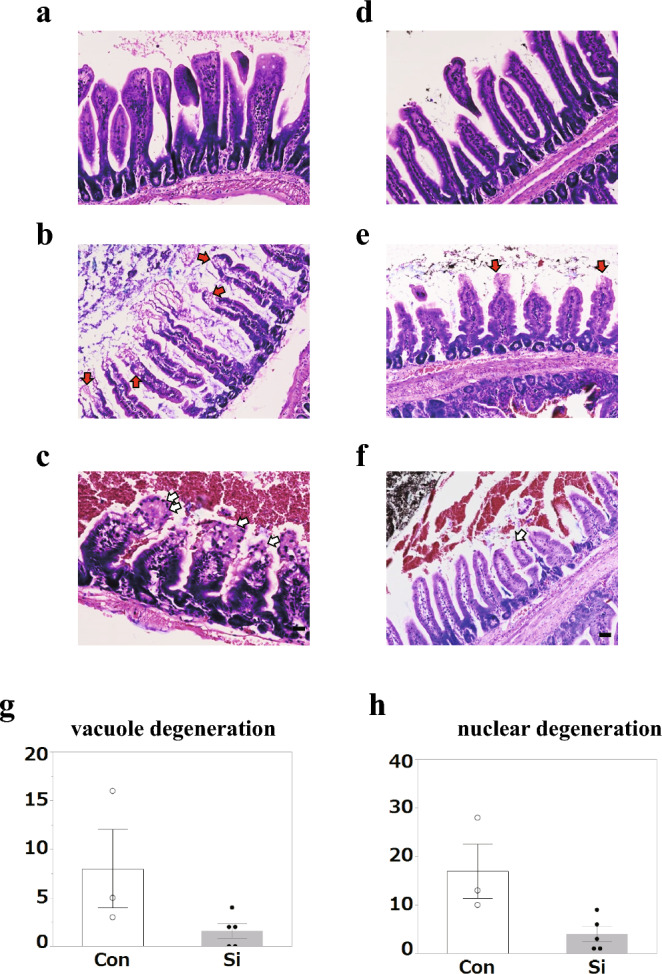
Pathological analysis of mouse small intestine using Giemsa-stained specimens. (**a**–**f**) The representative microphotographs of the Giemsa-stained small intestine in the control (Con) (**a**–**c**) and silicon-treated (Si) groups (**d**–**f**). (**a**, **d**) Normal site, (**b**, **e**) vacuole degeneration, and (**c**, **f**) nuclear degeneration. Red arrows: vacuole degeneration; White arrows: nuclear degeneration. Scale bar: 40 µm (**a**–**f**). (**g**, **h**) The measured results of the area of vacuole degeneration (**g**) or nuclear degeneration (**h**) in the small intestine. Bar chart indicates the mean values. White: Con group. Grey: Si group. Data are expressed as mean. Standard error of the mean (SEM) of three (Con) or five mice (Si) per group. *p < 0.05 versus Cont group, determined by Student’s paired *t*-test.

Subsequently, structural changes in the small intestine were observed in detail using histological Giemsa staining. Giemsa staining is a staining method used for blood smears and bone marrow specimens, but it has the characteristic of clearly staining the nuclei and degenerated vacuoles revealing their morphology and is also applied to tissue specimens (Fig. 2a, b)^18^. Tissue Giemsa staining revealed that the villous injury area with nuclear and vacuolar degeneration of absorptive epithelial cells was clearer than that observed with hematoxylin and eosin (HE) staining. Many regions where the luminal cells were unstained or lightly stained with HE were confirmed to be vacuolated by Giemsa staining (Fig. 2c, d). Furthermore, even in areas where the cytoplasm was stained up to the luminal side by HE, the nuclei of these cells were unstained with Giemsa solution (Fig. 2e, f). Unlike HE staining, Giemsa staining detected epithelial cells that had lost cell function, although the structure of the villi remained. Therefore, nuclear degeneration and vacuolation of the epithelial cells were used as indicators for analysis using tissue Giemsa staining. The ratio of the length of the epithelial cells that had undergone nuclear degeneration and vacuolation to the total length of the small intestine was determined (Fig. 2g, h). Statistical analysis showed that, compared with the Con group, the Si group showed a significant decrease in both vacuolation and nuclear degeneration.

### Si-based agent reduced the shedding of small intestinal goblet cells due to ischemia–reperfusion injury

**Figure 3:**
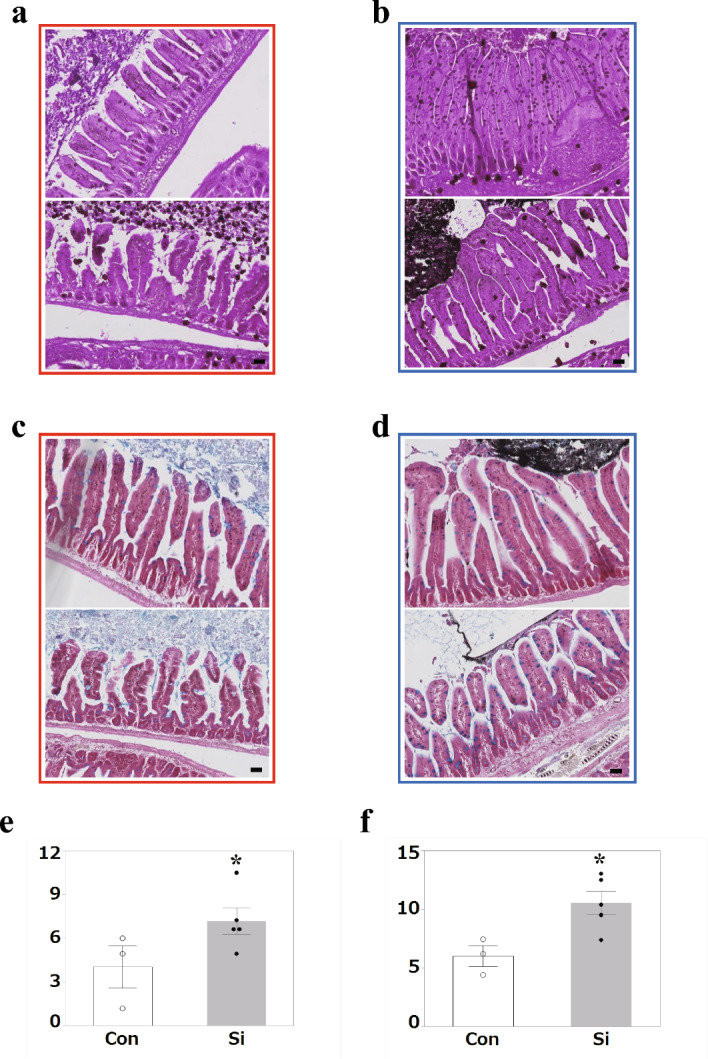
Examination of goblet cells by mucin staining. (**a**–**d**) The representative microphotographs of the periodic acid-Schiff (PAS)-stained (**a**, **b**) or the Alcian blue-stained (**c**, **d**) or small intestine in the control (Con) (**a**, **c**) and Si groups (**b**, **d**). Scale bar: 40 µm (**a**–**d**). (**e**, **f**) The measured results of number of goblet cells in the damaged area in the Alcian blue-stained (**e**) or the PAS-stained (**f**) small intestine. Bar chart indicates the mean values of Goblet cells per villi. White: Con group. Grey: Si group. Data are expressed as mean. Standard error of the mean (SEM) of three (Con) or five mice (Si) per group. *p < 0.05 versus Con group, determined by Student’s paired *t*-test.

Next, we examined whether mucus secretion persisted in the surviving non-injured area after IR. Two types of mucin staining, periodic acid-Schiff (PAS) staining (Fig. 3a, b) and Alcian blue staining (Fig. 3c, d), were performed on goblet cells responsible for mucin secretion in intestinal epithelial cells. PAS stains neutral mucins and Alcian blue stains acidic mucins. In the small intestinal tissue of normal mice, the secretory vesicles of the goblet cells scattered in the intestinal epithelial cells were mainly stained to fill the goblet cells. Morphological analysis revealed that the number of positive goblet cells was lower in the Con group than in the Si group (Fig. 3a–d). Statistical analysis showed that the number of positive goblet cells in the Si group was significantly higher than that in the Con group for both stained specimens (Fig. 3e, f).

### Si-based agent prevented cell death of small intestinal villi associated with ischemia–reperfusion injury by suppressing oxidative stress

**Figure 4:**
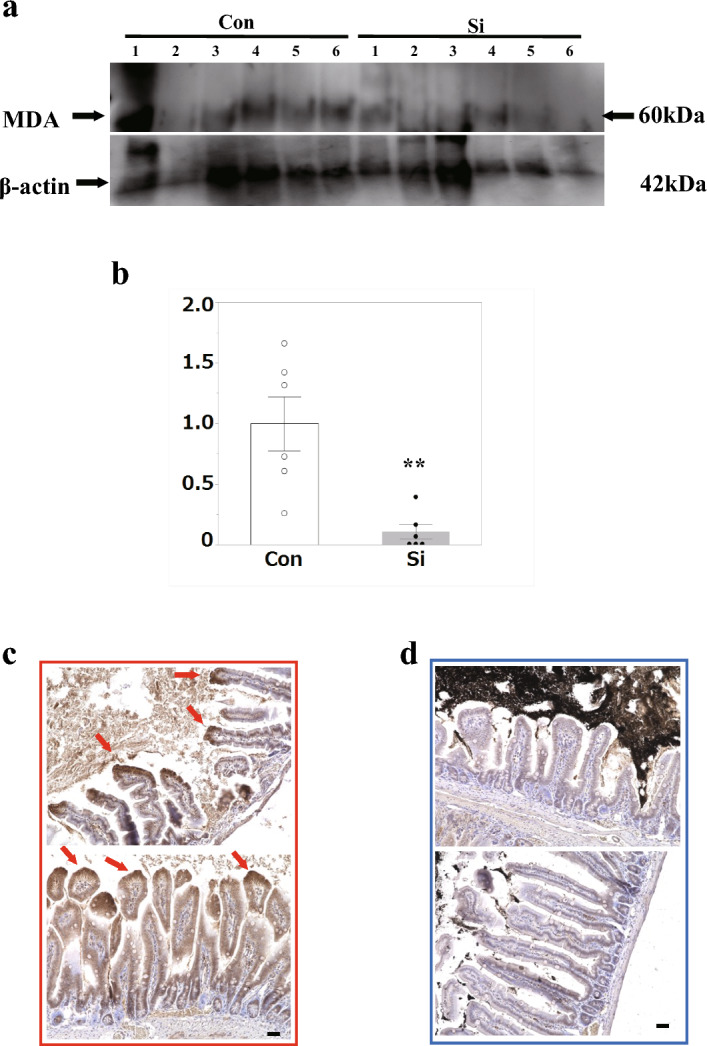
Oxidative stress analysis in small intestinal ischemia–reperfusion injury. (**a**) Western blot analysis of malondialdehyde (MDA) (60 kDa: upper panel) and β-actin (42 kDa: bottom panel) in the small intestine. (**b**) Bar graph indicating the densitometric quantification of bands. Data are expressed as mean. Standard error of the mean (SEM) of six mice per group. **p < 0.01 versus Control (Con) group, determined by Student’s paired *t*-test. (**c**, **d**) Immunofluorescence staining for 4-hydroxynonenal (4-HNE). The representative microphotographs of the small intestine in the small bowel ischemia–reperfusion injury mouse models. (**c**) Con group, (**d**) silicon-treated (Si) group. Red arrows: 4-HNE accumulation. Scale bar: 40 µm.

Furthermore, to investigate whether Si-based agent mitigates epithelial cell damage through their antioxidant effects, we analyzed lipid peroxides in the small intestine of each group. Western blotting was performed using an antibody against malondialdehyde (MDA), and the amount of lipid peroxide in the entire small intestine was compared. MDA is the final product of the oxidation of polyunsaturated fatty acids by ROS and reflects the degree of oxidative stress. The expression level of MDA was significantly suppressed in the Si group compared to that in the Con group (Fig. 4a, b). Subsequently, to investigate the accumulation of lipid peroxides in cells, we performed immunostaining using 4-HNE antibody, a marker of the lipid peroxide chain reaction. In the Con group, positive signals were observed in the cytoplasm of cells on the luminal side of the villi; however, in the Si group, few positive signals were observed (Fig. 4c, d).

**Figure 5:**
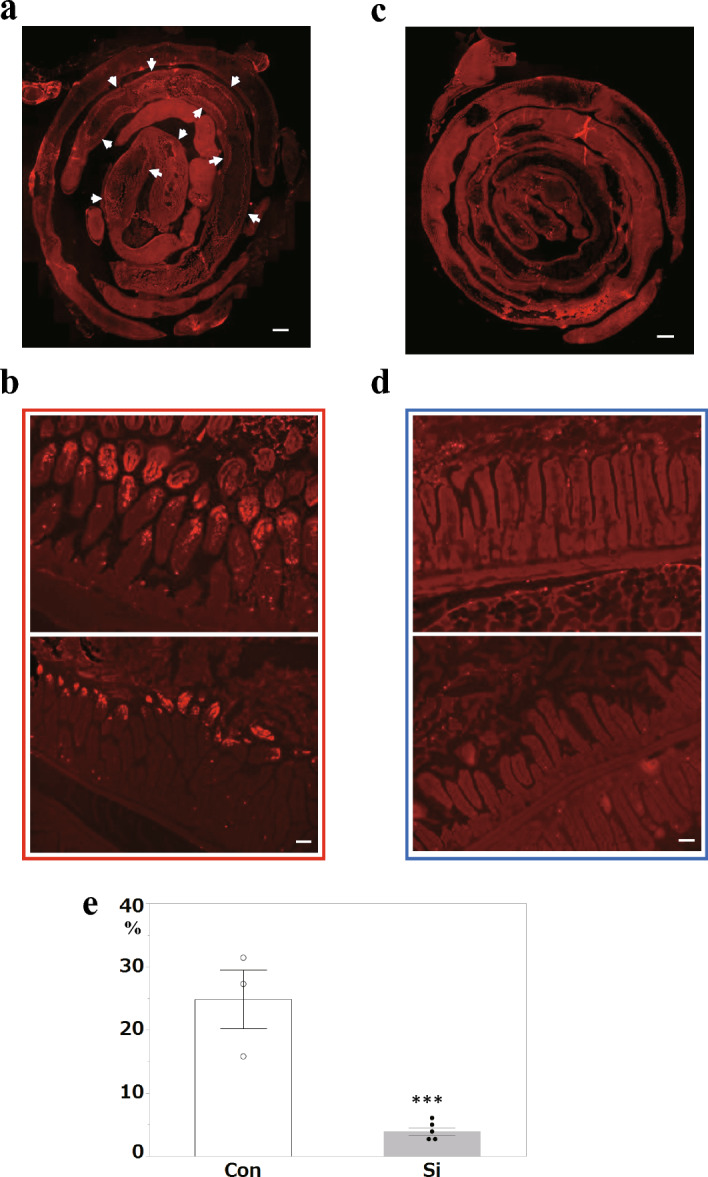
Apoptosis analysis in small intestinal ischemia–reperfusion injury. (**a**–**d**) The representative microphotographs of the hematoxylin and eosin (HE)-stained small intestine in the control (Con) group (**a**, **b**) and silicon-treated (Si) group (**c**, **d**). (**a**, **c**): Whole small intestine; (**b**, **d**): lesion site of the small intestine. White arrows: positive signals. Scale bar: 200 µm (**a**, **c**); 40 µm (**b**, **d**). (**e**) The measured results of the lesion area in the small intestine. Bar chart indicates the mean values. White: Con group. Grey: Si group. Data are expressed as mean. Standard error of the mean (SEM) of three (Con) or five mice (Si) per group. ***p < 0.001 versus Control group, determined by Student’s paired *t*-test.

Finally, considering that tissue damage caused by IR injury is the result of oxidative stress-induced cell death, we analyzed apoptotic cells using TUNEL staining. In the Con group, a wide range of positive signals were detected (Fig. 5a, b). In contrast, almost no positive signals were detected in the Si group (Fig. 5c, d). The apoptotic area was significantly decreased in the Si group compared to that in the Con group (Fig. 5e).

In conclusion, these results demonstrate that Si-based agents reduce the death of epithelial cells associated with small intestinal IR injury and significantly reduced tissue damage through antioxidant effects.

## Discussion

Small intestinal IR injury, a pathological condition that occurs due to mesenteric artery occlusion from thrombosis/embolism or small intestine transplantation. If the condition worsens, it may lead to a loss of intestinal function. Therefore, there is an urgent need to develop therapeutic agents that can prevent IR injury at early stages and suppress its progression. In this study, it was shown that an Si-based agent significantly suppressed cell damage, such as apoptosis, in small intestinal epithelial cells, which were composed of goblet and absorptive epithelial cells, thereby maintaining the mucus secretion ability of goblet cells. These findings indicate that Si-based agents could be effective therapeutic agents for small intestinal IR injury.

In the small intestine, the length of the villus generally shortens as it progresses from the rostral jejunum to the caudal ileum. Therefore, large differences in the length of the villi in the small intestine can be seen even in normal mice, depending on the site of specimen selection. Therefore, a region of villus that has shortened beyond a certain length should not be categorically determined as an injured region. When compared with adjacent villi, non-staining of the cytoplasm of intestinal epithelial cells and discontinuity between cells were observed. Areas where the villi were shortened and the structure was disrupted were defined as damaged areas. In addition, because of the differences in villus structure between the regions of the small intestine, only a portion of the small intestine should not be used for comparative analysis. To analyze the entire structure of the small intestine, a morphological analysis was performed using small intestine specimens prepared using the Swiss-Roll method. Taken together, our results accurately reflected the pathological changes in the entire small intestine and the effectiveness of Si-based agents.

In intestinal diseases, such as acute mesenteric artery occlusion and small bowel transplantation, in which blood flow is temporarily blocked, IR injury that occurs during revascularization is an inevitable complication. The main cause of IR injury is oxidative stress caused by ROS, which is generated when a large amount of oxygen is supplied to the hypoxic ischemic area during revascularization^6^. ROS impair mitochondrial function in cells, induce apoptosis, and cause organ damage. Furthermore, ROS generate lipid peroxides by oxidizing lipids such as phospholipids that constitute cell membranes and other polyunsaturated fatty acids. In recent years, lipid peroxide has been shown to have direct adverse effects on organs. Lipid peroxides caused by IR have been suggested to exacerbate IR injury through the activation of inflammatory response mechanisms such as the stimulator of interferon genes (STING) pathway^19^. In addition, increased levels of lipid peroxides impair not only the affected small intestine but also the function of remote organs such as the heart as a secondary disorder^20^, causing significant adverse effects on the whole body. Therefore, inhibition of lipid peroxide generation is of great importance in the treatment of IR injury in the small intestine.

Treatments with antioxidants, are effective in removing oxidants harmful to the body, such as lipid peroxide. Therefore, various studies have been conducted to suppress reperfusion injury by reducing the ROS (especially lipid peroxides) associated with IR. Specifically, superoxide dismutase (SOD) administration reduced IR injury^21^, and cell viability was improved in mice overexpressing Nrf-2, which increased the expression of antioxidant enzymes in response to oxidative stress^22^. In addition to the activation of the in vivo antioxidant system, the administration of antioxidants is also effective in suppressing IR injury. In particular, administration of polyphenols such as genistein^9^, resveratrol^10^, and oleuropein aglycon^11^ is effective. However, many polyphenols have a structure similar to that of estrogen, a female sex hormone, and there are concerns regarding side effects when administered at high doses ^23^. In particular, considering that acute mesenteric artery occlusion, which causes IR injury, frequently occurs in elderly people with a history of heart diseases such as arteriosclerosis and atrial fibrillation, the development of antioxidants with fewer side effects is required.

In contrast, hydrogen specifically scavenges hydroxyl radicals and is an excellent antioxidant with no reported adverse effects. It has been reported to exhibit anti-inflammatory effects^24^. However, the administration of hydrogen to living organisms is a matter of concern. When administered at a concentration sufficient to exhibit an antioxidant effect in the body, the conventional route of administration, oral or nasal inhalation, involves the risk of explosion due to the combustibility of hydrogen. Moreover, when orally administering hydrogen-dissolved water, the dissolved concentration is very low and cannot be maintained owing to its excellent permeability; therefore, it is not suitable for long-term storage. Our Si-based agent could be a solution to these problems. Si-based agents can react with water to generate large amounts of hydrogen continuously^13^. Upon oral administration in mice, an increase in the hydrogen concentration was observed in the mouse intestinal tract^15^. Thus, Si-based agents can stably and safely supply hydrogen, which is an excellent antioxidant, to the body. In addition to being effective against various diseases associated with oxidative stress, such as ulcerative colitis, chronic renal failure, interstitial pneumonia, and Parkinson’s disease^14,15,25^, Si-based agent has been reported to have symptom-suppressive effects on reperfusion injuries, such as skin flap IR injury and renal IR injury^16,17^.

In this study, we succeeded in mitigating the oxidative stress caused by small-intestinal IR by pre-administering an Si-based agent to constantly fill the intestinal tract with hydrogen. As mentioned above, it is important to suppress the production of lipid peroxides during small intestinal IR injury. 4-HNE and MDA are markers of the lipid peroxidation chain reaction and lipid peroxidation end products, respectively. Because Si-based agents significantly suppress the production of both lipid peroxides, they are considered to inhibit the chain reaction of lipid peroxides, thereby reducing the final accumulation of lipid peroxides and mitigating oxidative stress. Si-based agents inhibit apoptosis of small intestinal epithelial cells, including goblet cells, caused by oxidative stress. In conclusion, we suggest that Si-based agents may be effective therapeutic agents for small intestinal IR injuries.

Secretory mucin, the main component of mucus, is secreted from the epithelial cells of each organ, and is roughly divided into acidic and neutral mucins. In general, the distribution of these two types of mucin differs from organ to organ. Neutral mucins are present in the gastric mucosa^26^ and Brunner’s glands of the duodenum^27^, whereas acidic mucins are present in the colonic mucosa (particularly in the distal colon)^28^, and bronchial cords. However, there is no clear distribution of these two mucins in the small intestine. Goblet cells in the proximal small intestine secrete a high proportion of neutral mucins, whereas those in the distal small intestine secrete a high proportion of acidic mucins^29^. However, it is generally believed that neutral and acidic mucins coexist in the small intestine. The mucus layer containing these mucins plays a role in the physical defense against the invasion of digestive enzymes and intestinal bacteria, and it is important to maintain the mucus layer to maintain homeostasis of the intestinal environment. However, the pre-existing mucus layer is destroyed during early ischemia^30^. Therefore, maintaining the reserve capacity of goblet cells that can secrete new mucins when hemodynamics are re-established is of great importance in the treatment of small bowel IR injury. In this study, PAS staining for neutral mucins and Alcian blue staining for acidic mucins were used to evaluate the mucus secretion reserve of small intestinal goblet cells. Compared with the Con group, the Si group tended to have a higher number of staining-positive cells with PAS staining, and the number of staining-positive cells with Alcian blue staining was significantly higher. According to reports, the number of goblet cells per villus in normal mice is approximately 10; therefore, administration of Si-based agents almost completely suppresses the loss of goblet cells due to IR injury^31,32^. Therefore, it has been suggested that Si-based agents maintain their mucus-secreting ability by suppressing goblet cell dysfunction and protecting small intestinal epithelial cells from further damage. Furthermore, suppression of goblet cell reduction enables early recovery of the mucosal layer of mucin that is lost in ischemic conditions; therefore, it has also been suggested that Si-based agents may protect organs from late complications, such as infections and ulcers.

Because organ transplantations, such as those of the lungs, heart, and kidneys, are closely related to IR injury, the success rate of transplantation depends on the extent to which IR injury is suppressed^33,34^. According to previous reports, the administration of hydrogen gas has been proven effective in suppressing IR injury after liver and lung transplantation^35,36^, and hydrogen is very effective for successful organ transplantation^37,38^. Filling the lumen with hydrogen also protected the intestinal tract from IR injury after transplantation^39,40^. Considering these findings, Si-based agents may be therapeutic drugs that improve transplantation results, and future prospects for Si-based agents are anticipated.

## Materials and methods

### Small intestinal ischemia–reperfusion injury mouse model and diet

**Figure 6:**
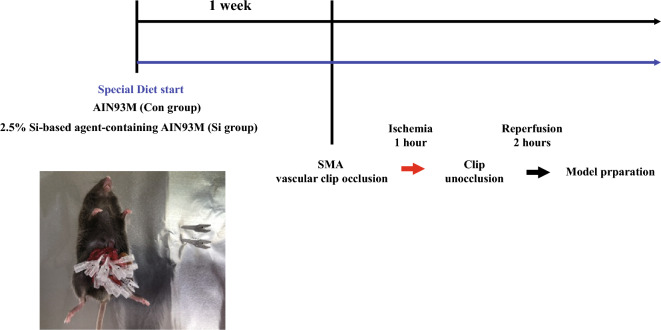
Experimental design. Bottom left diagram: a representative photo of SMA clip occlusion treatment. SMA: superior mesenteric artery.

Seven-week-old C57Bl/6 J male mice (Japan SLC, Shizuoka, Japan) were used in this study. The mice were reared at 23–25 °C and fed both special-order rodent chow and water ad libitum. Two customized rodent diets (Oriental Yeast Co., Ltd., Tokyo, Japan), AIN93M (control diet) and AIN93M containing 2.5% Si-based agent (Si-based agent-containing diet), were prepared as previously described^13–15^. The research design is illustrated in Fig. 6. We fed the mice each diet one week before IR treatment. Mice were randomly divided into the following two groups (12 mice per group): Con group, control diet-fed intestine-IR injury mouse model; and Si group, Si-based agent diet-fed small intestinal IR injury mouse model. Small-intestinal IR injury mouse models were prepared as described by Ekaterina et al. Under deep anesthesia using a combination anesthetic (0.3 mg/kg medetomidine, 4.0 mg/kg midazolam, and 5.0 mg/kg butorphanol)^41^, a laparotomy was performed approximately 4 cm in the midline using surgical scissors, and the jejunum was exposed by grasping the ileum with forceps and then pulling it outside the body. The mesentery was excised, and the superior mesenteric artery was identified without interference using microvascular clips (Bear Medic Co., Ibaraki, Japan). All primary branches of the superior mesenteric artery were clipped, and secondary branches were carefully clipped. After confirming that the blood flow in the area involving the superior mesenteric artery was completely blocked, the intestinal tract was maintained in an ischemic state for 1 h. Thereafter, all blood vessel clips were released, and the blood was reperfused for 2 h to induce small intestinal IR injury. We did one preliminary test and two main tests (3 mice/group/test). In a preliminary test (12 mice), we examined the number and position of microvascular clips during ischemia so that the extent of damage caused by ischemia–reperfusion would remain constant, and established conditions for preparing small intestinal IR injury mouse models. Two main tests were performed: various morphological analyses and Western blotting. In mouse rearing, since 3 mice/cage were used, one test was divided into 2 times, resulting in 6 mice per group. Including the preliminary test, the total number of mice used in the experiment is 36. Twelve mice per group in two main tests were used for analysis. Mice that died after reperfusion were exclude from analysis (Con: 3, Si:1).

### Paraffin section preparation

Under deep anesthesia, mice were perfused with 4% paraformaldehyde in 0.1 M phosphate buffer (PB: pH7.4). After removing the entire length of the small intestine from the jejunum to the ileum, the small intestine was rolled around the jejunum according to the Swiss-Roll method^42^, placed in a 3.5 cm dish, and then immersed in the same fixative. After stepwise dehydration with ethanol (70%, 80%, 90%, and 100%), the samples were embedded in paraffin (tissue preparation, T580, FALMA, Tokyo, Japan) using Clear Plus (FALMA). Seven micrometer thick paraffin-embedded small intestine sections were prepared using a microtome (RM2145, Leica Microsystems K.K, Tokyo, Japan) and then mounted on MAS-coated glass slides (Matsunami-glass, Osaka, Japan). The sides were stocked at 4 °C until use.

### Tissue staining

After deparaffinization, various tissue staining procedures were performed using small intestine sections as described below. All the stained slides were dehydrated using an ascending ethanol series and mounted with Entellan (Merck KGaA, Darmstadt, Germany). For Giemsa staining, samples were dehydrated with isopropanol, dried, and mounted. All samples were analyzed using a Keyence microscope (Keyence Corporation, Osaka, Japan).

#### Hematoxylin and eosin (HE) staining

The sample stained with the hematoxylin solution (FUJIFILM Wako Chemicals Corporation, Osaka, Japan) at 22 ± 2 °C for 5 min, washed with running tap water for 10 min, and then stained with eosin solution (FUJIFILM Wako Chemicals Corporation) at 22 ± 2 °C for 3 min.

#### Giemsa staining

Giemsa staining was performed as described previously^18^. The samples stained with the May–Grünwald’s stain solution (Muto Pure Chemicals, Tokyo, Japan) in methanol at 22 ± 2 °C for 30 min, and then Giemsa solution (Merck KGaA) in 0.067 M phosphate buffered saline (PBS) at 22 ± 2 °C overnight. The slides were washed with 1% acetic acid solution.

#### Alcian blue staining

The samples were stained using an Alcian blue staining solution kit (Diagnostic Biosystems, Pleasanton, CA, USA), according to the manufacturer’s instructions. Acidic mucopolysaccharides were stained blue with an Alcian blue solution. The nuclei were counterstained with nuclear fast. Each reaction time is as follows: Alcian blue solution (15 min at 37 °C); nuclear fast red solution (5 min at 22 ± 2 °C).

#### PAS staining

Samples were stained using a PAS staining solution kit (ScyTek Laboratories, Inc., Logan, UT, USA) according to the manufacturer’s instructions. Polysaccharides such as mucopolysaccharides were stained purple with Schiff’s solution. Each reaction time is as follows: periodic acid solution (5 min at 22 ± 2 °C) and Schiff’s solution (15 min at 22 ± 2 °C).

#### TUNEL staining

For TUNEL staining, the samples were stained with an in situ cell death detection kit (Roche Diagnostics Corporation, Basel, Switzerland) according to the manufacturer’s instructions. The slides were re-fixed with 4% PFA and air-dried. After rinsing in 0.1 M PBS, penetration treatment was conduct with 0.1% Triton-X, 0.1% sodium citrate in DW at 4 °C for 5 min. Slides were washed in 0.1 M PBS thoroughly, followed by treatment with reaction mixture at 37 °C for 1 h. Slides were washed in 0.1 M PBS several times. DNA fragments from apoptosis were stained red.

### Image analysis

For HE, Giemsa, and TUNEL staining, micrographs of the small intestine per individual were analyzed using Image J 1.52a software (National Institute of Health, Bethesda, MD, USA) to quantify the lesion area. HE staining was used to measure villus-deficient areas, tissue Giemsa staining was used to measure areas with vacuolization and nuclear degeneration, and TUNEL staining was used to measure areas with positive signals. The damaged area was measured using the following formula:

$$ Lesion \, area \, \left( \% \right) \, = \, \left\{ {length \, of \, damaged \, area} \right\}/\left\{ {total \, length \, of \, small \, intestine} \right\} \, \times 100. $$

For PAS or Alcian blue staining, micrographs of the small intestine per individual were analyzed using the Image J 1.52a software (NIH) to quantify the number of goblet cells in the lesion area.

### Western blotting

The small intestine (from the jejunum to the ileum) was removed under deep anesthesia, suspended in 2 mL Kaplan–Meier buffer (0.05 M Tris–HCl, 0.15 M NaCl, 10% glycerin and 1% NP-40) containing a protease inhibitor (Nacalai Tesque, Kyoto, Japan), and then ultrasonically disrupted using SONIFIER 250 advanced (BRASON Ultrasonics Corporation, Danbury, CT, USA) to prepare small intestine suspensions. The tissue suspension was centrifuged and the supernatant (protein solution) was collected. It was diluted with Kaplan–Meier buffer to a concentration of 1 μg/μL to prepare sample buffers for electrophoresis. Sample buffers (20 μl/well) and Protein Ladder (Nacalai Tesque, Kyoto, Japan) were applied to an sodium dodecyl sulfate- polyacrylamide gel electrophoresis gel, and electrophoresis (40 mA for 70 min) was performed. After electrophoresis, the proteins were transferred (10 V for 90 min) to a membrane (Merck KGaA, IPVH00010). Electrophoresis and transfer were confirmed by staining the gel with Coomassie Blue staining solution (Nacalai Tesque, Kyoto, Japan) and the membrane with Ponceau solution (Beacle, Inc. Kyoto, Japan). After treatment with blocking buffer, 5 mM TBS containing 0.05% tween-20, 5% skim milk (Nacalai Tesque, 31,149–75) at 22 ± 2 °C for 30 min, primary antibody reaction was performed using anti-MDA and anti-GAPDH (1:300; MAB374, Merck) antibody in blocking buffer at 4 °C overnight. The membranes were washed in 5 mM Tris-buffered saline (TBS, pH 7.4) containing 0.05% tween-20 (TBST) thoroughly, followed by secondary antibody reaction using horseradish peroxidase (HRP)-conjugated anti-mouse immunoglobulin G (IgG) antibody in TBST at 22 ± 2 °C for 1 h. Visualization was performed using an ECL solution kit (West Pico Plus; Thermo Fisher, Hercules, CA, USA) and bands were photographed using a ChemiDoc Touch MP imaging system (Bio-Rad Scientific Laboratories, Inc., Waltham, MA). The raw data from this analysis is available as supplementary material. The area of the band was measured using the Image J 1.52a software (NIH). Comparative analysis of MDA expression levels was performed using the value corrected with β-actin (MDA/β-actin).

### Immunohistochemistry

Immunohistochemistry was performed using paraffin-embedded samples as previously described^15^. After deparaffinization, the slides were subjected to antigen retrieval treatment with citrate buffer (95 °C for 5 min; 22 ± 2 °C for 20 min) and then rinsed in 0.01 M PBS. After treatment with 0.01 M PBS containing 0.3% Triton-X and 3% bovine serum albumin to inhibit non-specific staining and increase permeability to antibodies, the slides were incubated with anti-4-HNE mouse monoclonal antibody (Japan Institute for the Control of Aging, Nikken SEIL Co. Ltd., Shizuoka, Japan) in the above solution at 4 °C overnight. After washing in 0.1 M PBS, the samples were incubated with biotin-conjugated anti-mouse IgG antibody (4-HNE; Vector Laboratories, Inc. Burlingame, CA, USA) in 0.01 M PBS at 22 ± 2 °C for 30 min. The signal was amplified with avidin–biotin complex and then visualized with 50 mM TBS (pH 7.4) containing 1.25% 3,3′-diaminobenzidine (DAB: Merck KGaA, Darmstadt, Germany) and 0.75% hydrogen peroxide. After quenching with 50 mM TBS, the slides were counterstained with hematoxylin solution. Finally, all the stained samples were dehydrated and mounted using Entellan (Merck KGaA). All the samples were analyzed using a Keyence microscope (Keyence Corporation).

### Data and statistical analysis

Student’s *t*-test was used to compare the differences between the Si and Con groups. The results of the statistical analysis are expressed as mean values ± standard error of the mean (SEM) and considered significant at *p < 0.05 versus Con group.

### Study approval

All animal experiments were approved by the Animal Ethics Committee of Osaka University (approval number 01-072-011) according to the National Institute of Health Guide for the Care and Use of Laboratory Animals. This study was conducted in compliance with the ARRIVE guidelines. Every endeavor was made to minimize the number and suffering of laboratory animals. After treatment, the animals were meticulously observed, and an analgesic nonsteroidal anti-inflammatory agent (NSAID: ibuprofen; 5 mg/kg) was injected intraperitoneally when the animals displayed pain-like behaviour. Moreover, if experimental animals exhibited abnormalities or hypothetical humane endpoints (e.g., difficulty in feeding/watering, dyspnea, and self-mutilation, they were immediately euthanized by intraperitoneal injection of pentobarbital (200 mg/kg).

### Ethical approval

All animal experiments were approved by the Animal Ethics Committee of Osaka University (approval number 01–072-011) according to the National Institute of Health Guide for the Care and Use of Laboratory Animals. This study was conducted in compliance with the ARRIVE guidelines.

### Supplementary Information

Supplementary Information.

## Data Availability

All relevant data are within the paper and its Supplementary Information files.
