# Supplementary material for: Si-based agent alleviated small bowel ischemia–reperfusion injury through antioxidant effects
Source: Sci Rep. 2024 Feb 20;14:4141. doi: 10.1038/s41598-024-54542-7 (PMC10876940; doi:10.1038/s41598-024-54542-7)
Supplement: Supplementary file 1 — Supplementary Information. [file 41598_2024_54542_MOESM1_ESM.pdf]

MDA

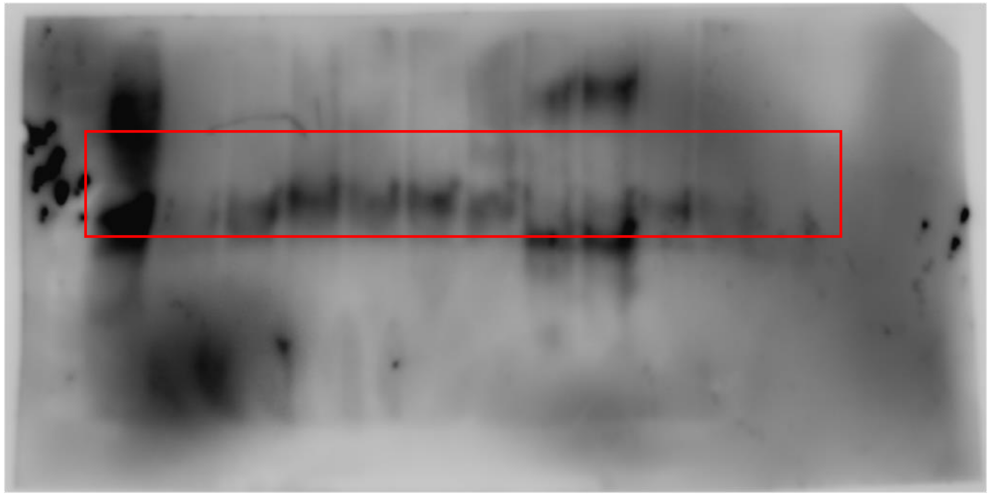

β-actin

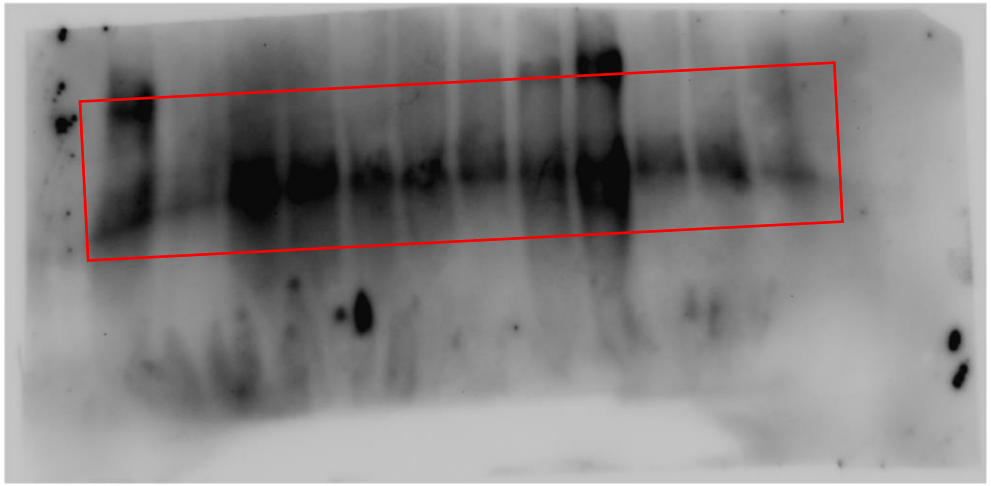

Overall photo of Western blotting gel in Figure 4a

MDA and β actin are run on different gels.

Red squares: the area used in Figure 4a
